# Disability and Self-care Living Strategies Among Adults Living With HIV During the COVID-19 Pandemic

**DOI:** 10.21203/rs.3.rs-868864/v1

**Published:** 2021-09-14

**Authors:** Kelly K O’Brien, Ahmed M Bayoumi, Soo Chan Carusone, Aileen M Davis, Rachel Aubry, Lisa Avery, Patty Solomon, Kristine M Erlandson, Colm Bergin, Richard Harding, Darren A Brown, Jaime H Vera, Steven Hanna

**Affiliations:** University of Toronto; University of Toronto; University of Toronto; University of Toronto; University of Toronto; University of Toronto; University of Toronto; University of Toronto; University of Toronto; University of Toronto; University of Toronto; University of Toronto; University of Toronto

**Keywords:** HIV/AIDS, COVID-19 pandemic, web-based survey, disability

## Abstract

**Background:**

Events associated with the COVID-19 pandemic, such as physical distancing, closure of community services, postponement of health appointments, and loss of employment can lead to social isolation, financial uncertainty, and interruption of antiretroviral adherence, resulting in additional health-related challenges (disability) experienced among adults living with chronic illness such as HIV. ‘Living strategies’ is a concept derived from the perspective of people living with HIV, defined as behaviors, attitudes and beliefs adopted by people living with HIV to help deal with disability associated with HIV and multi-morbidity. Our aim was to describe disability among adults living with HIV and self-care living strategies used during the COVID-19 pandemic.

**Methods:**

Adults living with HIV in Toronto, Ontario, Canada, including some with pre-pandemic HIV Disability Questionnaire (HDQ) data, completed a cross-sectional web-based survey between June-August 2020. The survey included the HDQ and questions about self-care living strategy use during the pandemic. We compared disability (HDQ) scores prior to versus during the pandemic using paired t-tests. We reported the proportion of participants who engaged in various living strategies at least ‘a few times a week’ or ‘everyday’ during the pandemic.

**Results:**

Of the 63 respondents, 84% were men, median age 57 years, and 62% lived alone. During the pandemic the greatest disability severity was in the uncertainty (median 30; Interquartile range (IQR): 16, 43) and mental-emotional (25; IQR: 14, 41) domains. Among the 51 participants with pre-pandemic data, HDQ severity scores were significantly greater (worse) during the pandemic (vs prior) in all domains. Greatest change from prior to during the pandemic was in the mental-emotional domain for presence (17.7; p<0.001), severity (11.4; p<0.001), and episodic nature (9.3; p<0.05) of disability. Most participants (>60%) reported engaging a ‘few times a week’ or ‘everyday’ in self-care strategies associated with maintaining sense of control and adopting positive attitudes and beliefs.

**Conclusions:**

People living with HIV reported high levels of uncertainty and mental-emotional health challenges during the pandemic. Disability increased across all HDQ dimensions, with the greatest worsening in the mental-emotional health domain. Results provide an understanding of disability and self-care strategy use during the COVID-19 pandemic.

## Introduction

Adults with HIV can experience complex physical, mental and social health-related consequences of multimorbidity, known as disability (1–7). Events associated with the COVID-19 pandemic, such as physical distancing, closure of community services, postponement of health appointments, and loss of employment can lead to social isolation, financial uncertainty, and interruption of antiretroviral adherence, further adding to disability for adults living with chronic illness such as HIV (8–10). Disability, in combination with health inequities, stigma, interrupted access to services, substance use, and food or home insecurity during the pandemic, can pose barriers to adults with HIV remaining engaged in care (11–14). During a pandemic, these stressors may exacerbate disability, placing individuals with HIV at risk of poorer adherence to antiretroviral therapy (15) and poor physical and mental health, associated with poorer retention in care (10, 16). However, the extent to which the pandemic may affect the severity and presence of disability among adults with HIV is unknown.

Due to measures implemented to slow the transmission of COVID-19, adults with HIV need to employ independent self-care living strategies in order to deal with uncertainty and the physical, social and mental health challenges of the pandemic (9, 13). Living strategies is a term that may be considered analogous to coping in the context of HIV. The concept of ‘living strategies’ was derived from the perspective of people living with HIV as a component of the Episodic Disability Framework, defined as behaviors, attitudes and beliefs adopted by people living with HIV to help deal with disability associated with HIV and multi-morbidity (7). Examples of living strategies include: seeking social interaction with others, maintaining a sense of control over life, blocking the event (i.e., the COVID-19 pandemic) out of the mind, and maintaining positive attitudes and beliefs (7). While most strategies have positive influences on health, some strategies may have negative health consequences, such as isolation (associated with physical distancing measures), or engaging in substance use. Understanding how these living strategies are used among adults living with HIV during this time can help to identify ways in which individuals and health care providers can promote timely and appropriate self-management approaches for enhancing health and well-being during a pandemic or other major events (17, 18).

Our aim was to describe disability experienced by adults with HIV and self-care living strategies used during the COVID-19 pandemic. Our primary objective was to describe the dimensions of disability experienced among adults with HIV during the pandemic and identify change in disability compared with prior to the pandemic. Our secondary objectives were to describe self-care living strategies used; and examine associations between disability and the frequency of living strategies use; and to identify changes in mental health and mastery compared with prior to the pandemic.

## Methods

### Study Design

We conducted a cross-sectional web-based survey using EQUATOR guidelines for web-based surveys (19, 20). This research was approved by the HIV/AIDS Research Ethics Board at the University of Toronto (Protocol #32910).

### Setting

In response to the World Health Organization’s declared outbreak of the novel coronavirus disease (COVID-19) the province of Ontario, Canada declared a state of emergency on March 17, 2020. To slow the transmission of SARS-CoV-2, the City of Toronto closed all shared, public or private recreational amenities and non-essential businesses, including community service organizations and health and fitness centres, and issued a stay-at-home order.

### Participants

Individuals were eligible if they self-identified as an adult ≥ 18 years living with HIV living in Toronto, Canada.

### Recruitment

We emailed adults with HIV who participated in a community-based exercise (CBE) intervention study (21) and agreed to be contacted about future research. Using a modified Dillman Tailored Design Method, we emailed potential participants an initial invitation and link to electronically administer, self-reported questionnaires using Qualtrics (22, 23). The email outlined the study purpose, eligibility, and involvement. Interested individuals opened a personalised Qualtrics link, first consented to participate by clicking yes to ‘I agree to participate in this research study’, and were the guided to the survey. We followed up with reminder emails at one, two and four weeks after the initial invitation. Participants received a $30 (Canadian dollar) electronic gift card for completing the survey. To increase our sample size, we asked participants to share details of the study with others living with HIV, who were asked to contact the research coordinator to review eligibility prior to enrolling in the study and receiving a personalized link to the survey.

## Data Collection

The survey included questionnaires capturing disability, self-care living strategies use, contextual factors (mental health, mastery), and COVID-19, health and demographic characteristics of participants. For participants recruited from the exercise study, we used HDQ data collected during that study (prior to the pandemic) to assess change in disability.

### Disability

We administered the HIV Disability Questionnaire (HDQ), to capture the presence, severity and episodic nature of disability experienced by adults aging with HIV across six domains including challenges with physical, mental-emotional, and cognitive health, day-to-day activities, social inclusion and uncertainty or worrying about the future (24, 25). The HDQ has demonstrated validity, reliability and sensibility for use with adults living with HIV (26–30). The HDQ captures uncertainty and the episodic nature of disability, making it an ideal tool in which to measure disability during the COVID-19 pandemic.

Self-Care Living Strategies: We administered a Living Strategies Questionnaire, adapted from categories in the Episodic Disability Framework and previously administered with adults with HIV in Canada (31). The 51-item questionnaire captured the frequency of living strategy use including: maintaining a sense of control in the context of uncertainty (lifestyle – physical activity, sleep health, nutrition, daily routine, re-establishing purpose, maintaining life balance, planning for and anticipating the future, and paying attention to health) (26 items); attitudes and beliefs (outlook on life, faith and spirituality) (8 items); blocking the COVID-19 pandemic out of the mind (7 items), and a section on seeking social interactions with others (10 items). For items 1–41, participants were asked how often they used a given living strategy in the past month ranging from 1 “None of the time (e.g. not at all)” to 4 “All of the time (e.g. every day)”. For the seeking social interaction items (Item 42–51), participants were asked how often they used a given social strategy in the past month ranging from 1 “None of the time (e.g. not at all)” to 5 “All of the time (e.g. every day)” or not applicable. For each, we asked whether the frequency of strategy use changed since the onset of the COVID-19 pandemic: increased (more frequent), decreased (less frequent) or no change.

### Personal Characteristics and COVID-19 Factors

We administered a demographic and COVID-19 questionnaire to capture personal characteristics (age, sex, gender, living status, income, employment status), HIV-related information (viral load, years since diagnosis, concurrent health conditions, self-reported health), and impact of the COVID-19 pandemic on access to health care services, supports, experiences and lifestyle changes.

Secondary Measures: *Mental Health*: The Patient Health Questionnaire (PHQ8) is an 8-item measure of depression severity (32) and possesses construct validity and test-retest reliability in adults with HIV (33, 34). *Mastery*: The Pearlin Mastery Scale includes 7 items that assess sense of personal control over life forces or outcomes (35). Each item in the scale is measured using a Likert scale with 4 response categories, and higher summary scores indicate greater levels of mastery.

## Analysis

We downloaded responses from Qualtrics for analysis using R (analytic analyses) (36). We calculated the view, participation and completion rates of survey responses (19). We calculated medians (IQR) and frequencies (%) to describe demographic characteristics of the sample. We conducted a cross sectional analysis with all participants who completed the web-based survey during the pandemic, and longitudinal analysis (prior to versus during the pandemic) among participants with pre-pandemic disability (HDQ), mental health (PHQ8) and mastery (mastery scale) data.

### Disability

We calculated medians and interquartile ranges (IQR) for HDQ presence, severity and episodic domain scores from all survey respondents. For participants recruited from the exercise study, we used paired t-tests to determine if their disability changed prior to versus during the pandemic. We used each participant’s median pre-pandemic HDQ score across all possible 12-time points in our previously 22 month exercise study (21) as the pre-pandemic score, and the current HDQ score from the web-based survey as the during pandemic score.

### Self-Care Living Strategies Use

We reported the frequency (%) of participants who engaged in each strategy ‘a few times a week’ or ‘everyday’ and change in strategy use (increase / decrease / no change). We conceptualized 37 of the items as having positive influences on health, 8 having negative influences on health, and 6 as having either a positive or a negative influence on health, dependent on the individual and context (31).

#### Secondary (exploratory) analyses:

We calculated median (IQR) scores for the PHQ8 (score range: 0–24) of which scores of ≥ 5, ≥10, and ≥ 20 indicate mild, moderate, and severe depression, respectively (32). We calculated median (IQR) for mastery scores, ranging from 7 to 28 with higher scores indicating greater levels of mastery (35). We calculated mean differences in pre and during pandemic PHQ8 (mental health) and mastery scores and conducted paired t-tests testing the null hypothesis that the mean change in disability score was zero (p < 0.05). We examined associations between HDQ domain severity scores during the pandemic (6 scores), and frequency of living strategies use (51 items) using Spearman correlational analysis for non-normally distributed data. We computed 306 rank correlations and used a Bonferroni adjusted alpha (*α_adj_* = 1.6 × 10^−4^) to report the most significant correlations. We chose the severity scale of the HDQ as this scale possesses most measurement properties.

## Results

We recruited participants and administered the web-based survey between June 9 and August 12, 2020. Of 114 exercise study participants emailed to participate in the study, 60 (53%) clicked on the survey link (view rate), 59/60 (98%) consented to participate and initiated the questionnaires (participation rate), and 51/59 (86%) consented and completed the questionnaires (completion rate) (19); and 12 additionally participated after word of mouth recruitment. Hence there was a total of 63 participants, 51 of which also had HDQ, PHQ8 and Mastery scale data collected prior to the pandemic.

### Characteristics of Participants and COVID-19 Factors

The majority of participants were men (84%), White (65%), single (54%), and living alone (62%) with a median age 57 years (IQR: 49, 65 years). No participants reported testing positive for COVID-19.

#### Health Care Services / Supports

Of the 61 (97%) participants who were receiving care from an HIV doctor or clinic, 71% received care remotely (telephone or online) during the COVID-19 pandemic (Table 1).

#### Experiences and Lifestyle Changes

Sixteen (25%) participants reported working during the pandemic. Among those not working, most (29/47; 62%) were not working prior to the COVID-19 pandemic. During the pandemic, participants reported boredom (79%), increased anxiety (75%) and depression (54%), and changes to sleep patterns (59%). Thirty-three (52%) reported accessing emotional or social support from friends, family, partners, or counselors (Table 1).

### Disability during the pandemic

HDQ scores are presented in Table 2. Across all 63 participants, the highest median HDQ severity, presence and episodic (daily fluctuations of health challenges the past week) scores were in the uncertainty, mental-emotional, and physical domains, respectively (Table 2).

Change in Disability (prior to versus during the pandemic)

**Figure 1:**
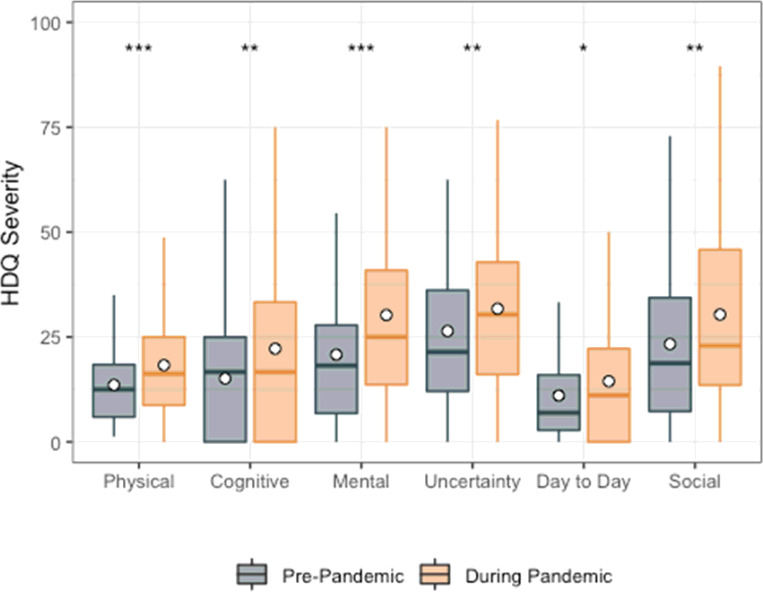
HDQ Severity mean domain scores prior to and during the pandemic (n=51). Significance of paired t-tests shown above each domain as follows: *** p<0.001, ** 0.001 < p ≤ 0.01, * 0.01< p ≤ 0.05. n=51 participants. Boxplots indicate median line and inter-quartile range (IQR), whiskers indicate 1.5 x IQR. Mean is shown as a circle.

Among the 51 participants with pre-pandemic HDQ scores, mean HDQ severity scores were significantly higher (greater disability) during compared to prior to the pandemic, for all domains. The largest increase in disability severity was in the mental-emotional domain (mean HDQ change score: 11.4; sd: 15.3) (Fig. 1; Table 3).

### Living Strategies

The majority of participants (> 60%) reported engaging in positive living strategies a ‘few times a week’ or ‘everyday’ pertaining to maintaining a sense of control (represented in 14 different strategies in areas of maintaining a healthy lifestyle, maintaining health as focus and purpose in life, maintaining life balance, planning for and anticipating the future, and paying attention to viral load to remain on top of health) and adopting positive attitudes and beliefs (represented by 5 different strategies related to positive outlook) (Additional File 3). Nine strategies (18%) changed since the onset of the pandemic by the majority of participants (> 50%), most of which pertained to social interaction (decreasing time spent with friends, colleagues or seeking company with others, increasing isolation, and increasing time interacting with others on the internet). Forty-eight percent of participants reported increasing their use of interacting with others on the internet, and 27% engaged in this strategy ‘a few times a week’ or ‘everyday’ (Additional File 3).

### Associations between Disability and Self-Care Living Strategies during the COVID-19 Pandemic

All domains of the HDQ except the physical domain were significantly associated (Spearman rho | ≥ 0.46|) with frequency of use of eight living strategies. Maintaining a sense of control (focusing on things such as work, friends and activities (Spearman rho | 0.47|), maintaining a good balance of activity in life (Spearman rho | 0.51|), and trying to stick to daily structure or routine (Spearman rho | 0.46|), were associated with lower HDQ (improved) severity scores for mental-emotional health challenges. Living strategies pertaining to attitudes and beliefs (considering self to be healthy (Spearman rho | 0.50 to 0.60|), accepting and valuing who I am (Spearman rho | 0.47 to 0.52|), positive outlook on life (Spearman rho | 0.46 to 0.54|), choosing to believe one can overcome any challenges (Spearman rho | 0.47 to 0.53|), were associated with lower (improved) HDQ severity scores for challenges with mental-emotional and cognitive health, uncertainty, day-to-day activities, and social inclusion, and feeling hopeless (Spearman rho | 0.46 to 0.62|) was associated with greater (worse) HDQ severity scores for challenges with mental-emotional and cognitive health, and social inclusion (Additional File 4).

## Discussion

Using a previously validated disability questionnaire with adults with HIV administered prior to the pandemic, we found that self-reported disability severity increased across all dimensions during the COVID-19 pandemic among adults with HIV, with the largest increase found in the presence, severity, and episodic components of mental-emotional health. This finding was supported by the significant increase in mean PHQ8 scores and 39% reporting a higher category of depression during the pandemic compared to prior (Additional File 1 and 2). Similarly, the majority of participants reported ‘more anxiety’, ‘more depression’, or ‘frustration of boredom’ during the pandemic (Table 1).

Uncertainty followed by mental-emotional health challenges were the most severe dimensions of disability experienced during the pandemic (Table 2). These dimensions of disability are closely related as uncertainty is a strong predictor of mental-emotional health challenges for adults with HIV (37–39). The increase in uncertainty scores during the pandemic were supported by participants who reported fear of getting COVID-19 (75%), worrying about friends, family and partner (86%) (Table 1), suggesting factors that may be contributing to disability dimensions of uncertainty and mental-emotional health challenges in the HDQ scores. The concept of uncertainty in COVID-19 has been explored specifically as it pertains to diagnostic uncertainty and unknown severity, duration, and long-term impact of symptoms associated with COVID-19 (40–42). The impact of uncertainty associated with an ongoing pandemic, uncertainty of acquiring COVID-19 and subsequently Long COVID, and ongoing safety measures and policy among individuals health and well-being during the pandemic is unclear. Uncertainty increased in severity but not presence, suggesting that existing uncertainty was exacerbated by the pandemic. Adults with HIV may have prior experiences living with uncertainty from the earlier days of the HIV epidemic (38, 39, 43). However, the extent to which these prior experiences may exacerbate or equip an individual with strategies to deal with uncertainty during a new (COVID-19) pandemic is unknown and an area of future research.

In addition to the greater risk and poorer outcomes of COVID-19 that may occur among people with HIV (44–47), the pandemic can further exacerbate stress and isolation, unemployment, food insecurity, access to care, and difficulty managing underlying comorbidities (9, 48). These factors may amplify disability, as reflected by the increase of HDQ severity scores across all dimensions in the sample. Evidence is emerging on the impact of HIV and COVID-19 as co-pandemics, including the impact of co-infection on health services (14). Further qualitative inquiry may help to explore the nature and impact of the pandemic on health outcomes for adults with HIV.

The greatest change in disability was in the mental-emotional health domain, where participants experienced the largest increases in presence, severity and episodic scores of disability compared with pre-pandemic scores. Physical distancing measures and public health restrictions during the COVID-19 pandemic have been linked to loneliness and social isolation among adults with HIV (49), which may have exacerbated mental and emotional health challenges and challenges to social inclusion. Most participants were practising social distancing, reflected in the most common changes in living strategies related to social interaction. While 48% of participants reported increasing their use of interacting with others on the internet, only 27% frequently engaged in this strategy (Additional File 3). Given the majority of participants lived alone, this may further highlight challenges to social inclusion and mental-emotional health experienced during the pandemic. COVID-19 restrictions may additionally pose difficulties for attending medical appointments, or accessing medications, subsequently affecting the health of adults living with HIV (9, 10, 13). While most participants remained engaged in care by accessing their HIV doctor remotely (71%), some reported difficulties accessing (40%) and using (37%) health care services (Table 1). Of note, the pandemic appeared to have less of an impact on employment, which is likely a reflection of almost half of the participants (46%) who were not working prior to the pandemic.

The majority of participants reported sustained use of positive living strategies during the pandemic with the exception of positive social interaction strategies and negative strategies (e.g. isolating self) that increased during the pandemic (Additional File 3). Another study similarly reported that higher levels of social support and resilient coping were associated with lower depressive symptoms among adults with HIV in the context of the COVID-19 pandemic (50). The increase in social isolation (spending less time with support networks, less time going out or spending time with others, and tending to isolate self more), a likely consequence of the COVID-19 restrictions, aligns with the increase in disability in dimensions of social inclusion and mental-emotional health as measured by the HDQ. This highlights the role for remote social support and services that can promote social interaction and support among community during the pandemic.

To our knowledge this is the first study to explore the nature, extent and impact of disability experienced by adults with HIV prior to and during the COVID-19 pandemic, and to specifically explore the impact of the COVID-19 pandemic on health outcomes and living strategies use for people with chronic illness. This work provides insights to the health challenges experienced by adults with HIV who may be living with the added complexity of concurrent health conditions (51–53), and specifically the common mental health conditions such as anxiety and depression. Opportunities exist for health providers to apply the lessons learned in HIV rehabilitation to the context of disability experienced during the COVID-19 pandemic, such as anticipating and preparing for the impact of disability, acknowledging the episodic nature in which disability may be experienced, recognizing the impact of stigma and health inequities, understanding uncertainty in COVID-19 related disability, and implementing disability and rehabilitation-focused responses that include people affected by the COVID-19 pandemic that may help health providers better address the needs of people living with HIV during the COVID-19 pandemic (43, 54, 55).

We administered study questionnaires in-between a first (Spring 2020) and second (Fall 2020) wave of the COVID-19 pandemic in Toronto, Ontario. With COVID-19 restrictions continuing with third and fourth waves in Spring and Summer 2021, the sustained and potential cumulative impact of COVID-19 on disability dimensions are unclear. The clinical importance of HDQ change scores should be interpreted with caution. Pre-pandemic measures were collected from 2016–2018, hence it is unknown to what extent changes may be attributed to other contextual factors, such aging over time.

While a strength of our study was building on data collected pre-pandemic (HDQ, PHQ8, mastery) with an existing cohort of adults living with HIV who participated in an earlier exercise intervention study, our study is not without limitations. We assessed disability and living strategies use cross-sectionally during the pandemic, as distinct constructs and cannot infer causation between the pandemic and disability and living strategies use. Due to our small sample size, our analysis was exploratory. Furthermore, the clinical importance of HDQ change scores, particularly given the episodic nature of disability in the context of the COVID-19 pandemic is unclear. Nevertheless, our inclusion of supplemental measures of mental health (PHQ8) support the direction of change in disability this study. Given the majority of the sample were from a previous study examining the impact of community-based exercise among adults living with HIV, primarily including White men living in an urban setting, results may not be representative of the larger HIV population. Men and women can experience disability differently pertaining to hospitalization, parental roles, fatigue, mental health, and social inclusion (56–58). Future work should examine the influence of gender on the disability experiences among adults with HIV in the context of the pandemic.

## Conclusions

Participants with HIV reported high levels of uncertainty and mental health challenges during the pandemic. All six dimensions of disability increased during the pandemic, the largest increase was the mental-emotional health domain. Strategies involving maintaining a sense of control, and positive attitudes and beliefs were associated with lower disability during the pandemic. Results help to provide an understanding of disability and self-care living strategy use among adults living with HIV during the COVID-19 pandemic.

## Supplementary Material

Supplement 1

Supplement 2

Supplement 3

Supplement 4

## Figures and Tables

**Figure 2:**
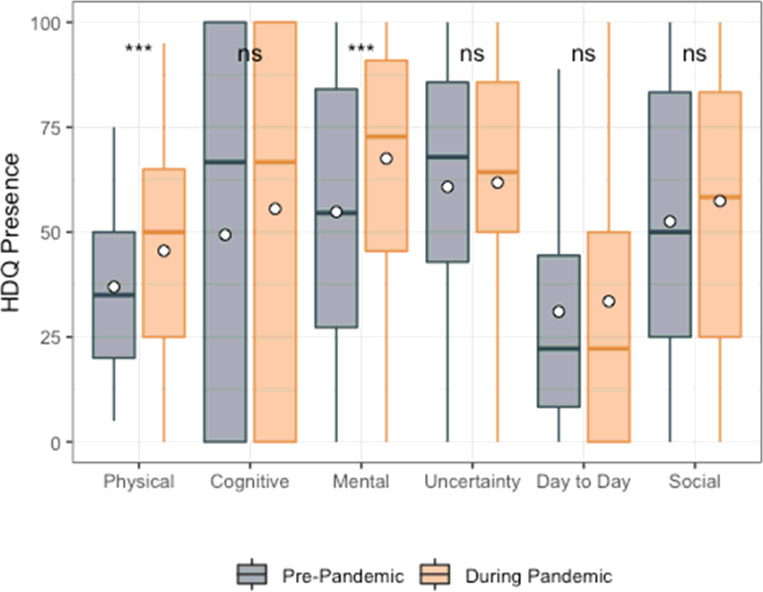
HDQ Presence mean domain scores prior to and during the pandemic (n=51). Significance of paired t-tests is shown above each domain as follows: *** p<0.001, ** 0.001 < p ≤ 0.01, * 0.01< p ≤ 0.05 and ns indicates p>0.05. n=51 participants. Boxplots indicate median line and inter-quartile range (IQR), whiskers indicate 1.5 x IQR. Mean is shown as a circle.

**Figure 3:**
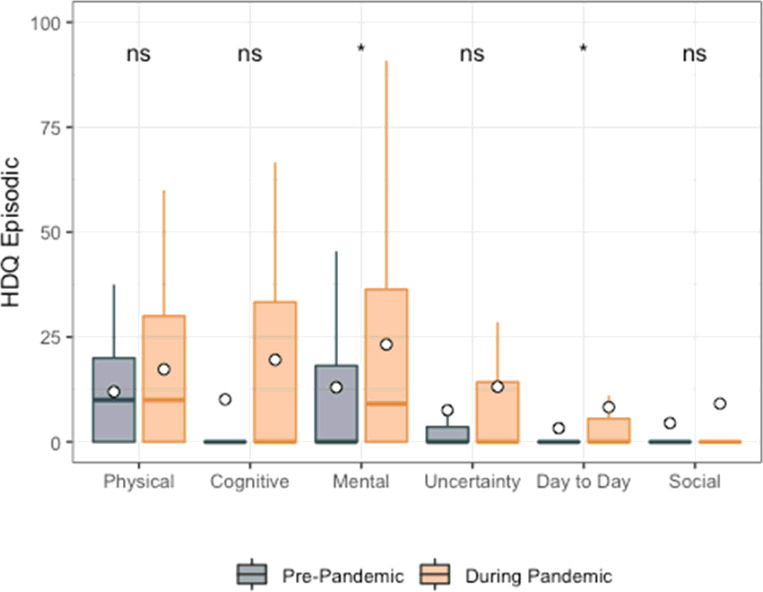
HDQ Episodic mean domain scores prior to and during the pandemic (n=51). Significance of paired t-tests is shown above each domain as follows: *** p<0.001, ** 0.001 < p <= 0.01, * 0.01< p <= 0.05 and ns indicates p>0.05. n=51 participants. Boxplots indicate median line and inter-quartile range (IQR), whiskers indicate 1.5 x IQR. Mean is shown as a circle.

**Table 1 –:** Characteristics of Participants, and COVID-19 Pandemic Health and Support Services, Experiences and Lifestyle Changes (n = 63)

Characteristics	Number (%)

**Median Age** (IQR)	57 years (49, 65)

**Sex**	53 (84%)
Male	10 (16%)
Female	

**Gender**	53 (84%)
Man	9 (14%)
Woman	1 (2%)
Non-Binary	

**Live alone**	39 (62%)

**Personal gross yearly income** <$30,000 CAD	33 (52%)

**Employment status**	18 (29%)
Working for pay	12 (19%)
Retired	18 (29%)
On Disability	

**Most Common Concurrent Conditions** (≥ 30%)	24 (38%)
Mental Health (e.g. depression, anxiety)	21 (33%)
Chronic pain (e.g. arthritis)	22 (35%)
Trouble sleeping (e.g. insomnia)	

**Undetectable viral load (< 50 copies/mL)** *	61 (97%)

**Median Number of Years since HIV Diagnosis (IQR)**	22 (11, 32)

**Overall Self-Reported Health** - Very good or Good	42 (67%)

**Health status compared to previous year**:	19 (30%)
Better now than one year ago	25 (40%)
About the same as one year ago	19 (30%)
Worse now than one year ago	

**COVID-19 Pandemic – Health Care Services / Supports**	**Number (%)**

**HIV Care**	45 (71%)
Remote (telephone / online) connect with HIV doctor or clinic during the pandemic	10 (16%)
Access to HIV medication impacted by pandemic	25 (40%)
Difficulties accessing healthcare	23 (37%)
Decreased health care services use during COVID	

**COVID-19 Pandemic – Experiences and Lifestyle Changes**	**Number (%)**

Working during COVID-19	16 (25%)
Not working prior to pandemic (out of the 47 not working)	29 (62%)
Reduced wages or work hours	22 (35%)
Employment status changed since COVID-19 pandemic	20 (32%)

**COVID-19 Experiences**	33 (52%)
Getting support from friends, family, partners, etc.	47 (75%)
Fear of getting COVID-19	35 (56%)
Fear of giving COVID-19 to someone else	54 (86%)
Worrying about friends, family, partner, etc.	50 (79%)
Frustration of boredom	47 (75%)
More anxiety	34 (54%)
More depression	37 (59%)
Changes to sleep patterns	34 (54%)
Alcohol use in past 30 days	24 (38%)
Cannabis use in past 30 days	

**Lifestyle changes due to COVID-19 pandemic**	60 (95%)
Practicing social distancing	44 (70%)
Isolating / quarantining from others	54 (86%)
Avoiding going to gym or exercise class	55 (87%)
Following media coverage on COVID	44 (70%)
Changing travel plans	36 (57%)
Stocking up on food and supplies	46 (73%)
Exercising less (frequency or intensity)	14 (22%)
Increase (frequency and amount) in substance use	

N = 63 participants’

* all participants were taking antiretroviral therapy medications

∼ remote includes via telephone or video.

**Table 2 –:** HIV Disability Questionnaire (HDQ) scores during the pandemic (n = 63 participants)

HDQ Domain	Presence Median (IQR)	Severity Median (IQR)	Episodic Median (IQR)
**Physical Symptoms**	50 (25, 65)	16 (9, 25)	10 (0, 30)
**Cognitive Symptoms**	66 (0, 100)	17 (0, 33)	0 (0, 33)
**Mental-Emotional Health Symptoms**	73 (46, 91)	25 (14, 41)	9 (0, 36)
**Uncertainty or Worry about the Future**	64 (54, 86)	30 (16, 43)	0 (0, 14)
**Difficulties with Day-to-Day Activities**	22 (0, 56)	11 (0, 25)	0 (0, 11)
**Challenges to Social Inclusion**	58 (25, 83)	23 (14, 48)	0 (0, 0)

LEGEND: HIV Disability Questionnaire (HDQ) score range; 0–100; higher scores indicate greater presence, severity and episodic nature of disability; n = 63 participants

**Table 3:** HDQ Severity Scores: Pre versus During COVID-19 Pandemic (n = 51)

HDQ Domain	Spearman Correlation coefficients	Pre-Pandemic Mean (sd) HDQ Severity Scores	During Pandemic Mean (sd) HDQ Severity Scores	Mean (sd) Change in HDQ Severity Scores∼	Paired T-test	P value
**Physical**	0.6	13.6 (9.3)	18.5 (12.0)	4.9 (9.6)	−3.64	0.001***
**Cognitive**	0.6	15.1 (14.9)	21.9 (22.3)	6.8 (17.6)	−2.76	0.008*
**Mental-Emotional**	0.7	20.8 (18.0)	32.2 (21.3)	11.4 (15.3)	−5.30	< 0.001***
**Uncertainty**	0.8	26.4 (20.3)	31.1 (22.0)	4.7 (11.5)	−2.93	0.005**
**Day-to-Day Activities**	0.7	11.1 (11.9)	15.1 (18.5)	4.1 (11.9)	−2.45	0.018*
**Social Inclusion**	0.8	23.3 (18.7)	29.2 (23.0)	5.8 (14.2)	−2.93	0.005**

LEGEND: HDQ severity scores range: 0–100; degrees of freedom: 50

∼ Mean change score (during – prior to pandemic for each participant).

*** indicates p ≤ 0.001

** indicates 0.001 < p ≤ 0.01

* indicates 0.01 < p ≤ 0.05.

Among HDQ presence scores, only physical (mean HDQ change score: 10.3; sd: 20.7) and mental-emotional (mean HDQ change score: 17.7; sd: 26.2) were significantly higher (greater presence of disability) during compared to prior to the pandemic (Fig. 2; Table 4).

**Table 4:** HDQ Presence Scores: Pre versus During COVID-19 Pandemic (n = 51)

HDQ Domain	Spearman Correlation coefficients	Pre-Pandemic Mean (sd) HDQ Presence Scores	During Pandemic Mean (sd) HDQ Presence Scores	Mean (sd) Change in HDQ Presence Scores	Paired T-test	P value
**Physical**	0.6	37.0 (20.9)	47.3 (24.8)	10.3 (20.7)	−3.56	0.001***
**Cognitive**	0.6	49.3 (41.6)	56.2 (42.9)	6.9 (37.9)	−1.29	0.202
**Mental-Emotional**	0.6	54.8 (31.6)	72.5 (25.9)	17.7 (26.2)	−4.84	< 0.001***
**Uncertainty**	0.8	60.8 (29.4)	61.8 (26.3)	1.0 (17.3)	−0.41	0.687
**Day-to-Day Activities**	0.6	31.0 (28.0)	36.2 (34.1)	5.1 (26.0)	−1.41	0.166
**Social Inclusion**	0.8	52.5 (31.3)	56.7 (30.6)	4.2 (18.7)	−1.59	0.118

LEGEND: HDQ presence scores range: 0–100; degrees of freedom: 50; *indicates significant change score p < 0.05

*** indicates p < 0.001

** indicates 0.001 < p < = 0.01

* indicates 0.01 < p < = 0.05.

Among HDQ episodic scores, only mental-emotional (mean HDQ episodic change score: 9.3; sd: 30.6) and difficulties carrying out day-to-day activities (mean HDQ episodic change score: 5.2; sd: 16.2) were higher during compared to prior to the pandemic (Fig. 3; Table 5).

**Table 5:** HDQ Episodic Scores: Pre versus During COVID-19 Pandemic (n = 51)

HDQ Domain	Spearman Correlation coefficients	Pre-Pandemic Mean (sd) HDQ Episodic Scores	During Pandemic Mean (sd) HDQ Episodic Scores	Mean (sd) Change in HDQ Episodic Scores	Paired T-test	P value
**Physical**	0.5	12.0 (14.3)	16.7 (18.9)	4.7 (18.9)	−1.78	0.082
**Cognitive**	0.5	10.1 (23.1)	17.6 (33.6)	7.5 (29.5)	−1.82	0.075
**Mental-Emotional**	0.4	13.0 (22.6)	22.3 (30.5)	9.3 (30.6)	−2.17	0.035*
**Uncertainty**	0.4	7.6 (18.4)	10.9 (19.7)	3.4 (16.0)	−1.50	0.140
**Day-to-Day Activities**	0.4	3.3 (8.8)	8.5 (18.4)	5.2 (16.2)	−2.30	0.026*
**Social Inclusion**	0.4	4.5 (14.9)	5.6 (17.7)	1.1 (13.7)	−0.55	0.582

LEGEND: HDQ episodic scores range: 0–100; degrees of freedom: 50

*** indicates p < 0.001

** indicates 0.001 < p < = 0.01

* indicates 0.01 < p < = 0.05.

*Secondary Measures*: Mental health (PHQ8) scores increased from 6.2 (pre-pandemic) to 8.0 (during the pandemic), dassified as ‘mild depression’ at both time points (change score: 1.9 points; p = 0.01) (Additional File 1). Across all depression categories of the PHQ8, 20 of the 51 participants (39%) reported increased depression during the pandemic, 6 (12%) reported less depression, and 25 (49%) remained stable (Additional File 2). There was no change in mastery scores (Additional File 1).

## Abbreviations

HIV: Human Immunode ciency Virus
HDQ: HIV Disability Questionnaire
IQR: Interquartile Range
PHQ8: Patient Health Questionnaire
SD: Standard Deviation

## References

[1] Antiretroviral Therapy Cohort Collaboration. Survival of HIV-positive patients starting antiretroviral therapy between 1996 and 2013: a collaborative analysis of cohort studies. The lancet HIV. 2017;4(8):e349–e56. 28501495 10.1016/S2352-3018(17)30066-8 PMC5555438

[2] Deeks SG, Lewin SR, Havlir DV. The end of AIDS: HIV infection as a chronic disease. Lancet. 2013;382(9903):1525–33. Epub 2013/10/25. 24152939 10.1016/S0140-6736(13)61809-7 PMC4058441

[3] Guaraldi G, Silva AR, Stentarelli C. Multimorbidity and functional status assessment. Current opinion in HIV and AIDS. 2014;9(4):386–97. Epub 2014/05/29. 24867388 10.1097/COH.0000000000000079

[4] Kendall CE, Wong J, Taljaard M, Glazier RH, Hogg W, Younger J, A cross-sectional, population-based study measuring comorbidity among people living with HIV in Ontario. BMC public health. 2014;14(1):161. Epub 2014/02/15. 24524286 10.1186/1471-2458-14-161 PMC3933292

[5] Erlandson KM, Schrack JA, Jankowski CM, Brown TT, Campbell TB. Functional impairment, disability, and frailty in adults aging with HIV-infection. Curr HIV/AIDS Rep. 2014;11(3):279–90. 24966138 10.1007/s11904-014-0215-y PMC4125474

[6] O’Brien KK, Bayoumi AM, Strike C, Young NL, Davis AM. Exploring disability from the perspective of adults living with HIV/AIDS: development of a conceptual framework. Health Qual Life Outcomes. 2008;6:76. Epub 2008/10/07. 18834538 10.1186/1477-7525-6-76 PMC2572592

[7] O’Brien KK., Davis AM., Strike C., Young NL., Bayoumi AM. Putting episodic disability into context: a qualitative study exploring factors that influence disability experienced by adults living with HIV/AIDS. J Int AIDS Soc. 2009;12(1):5. Epub 2009/11/11. 10.1186/1758-2652-12-30 PMC2788343 19900284

[8] Juni P, Rothenbuhler M, Bobos P, Thorpe KE, da Costa BR, Fisman DN, Impact of climate and public health interventions on the COVID-19 pandemic: A prospective cohort study. CMAJ : Canadian Medical Association journal = journal de l’Association medicale canadienne. 2020. Epub 2020/05/10. 10.1503/cmaj.200920 PMC7259972 32385067

[9] Shiau S, Krause KD, Valera P, Swaminathan S, Halkitis PN. The Burden of COVID-19 in People Living with HIV: A Syndemic Perspective. AIDS Behav. 2020. Epub 2020/04/19. 10.1007/s10461-020-02871-9 PMC7165075 32303925

[10] Jiang H, Zhou Y, Tang W. Maintaining HIV care during the COVID-19 pandemic. The lancet HIV. 2020;7(5):e308–e9. Epub 2020/04/10. 32272084 10.1016/S2352-3018(20)30105-3 PMC7239666

[11] Hull MW, Wu Z, Montaner JS. Optimizing the engagement of care cascade: a critical step to maximize the impact of HIV treatment as prevention. Current opinion in HIV and AIDS. 2012;7(6):579–86. Epub 2012/10/19. 23076123 10.1097/COH.0b013e3283590617

[12] Hoffmann M, MacCarthy S, Batson A, Crawford-Roberts A, Rasanathan J, Nunn A, Barriers along the care cascade of HIV-infected men in a large urban center of Brazil. AIDS Care. 2016;28(1):57–62. 26291264 10.1080/09540121.2015.1062462 PMC5082135

[13] Hargreaves J, Davey C, Group for lessons from pandemic HIVpftC-r. Three lessons for the COVID-19 response from pandemic HIV. The lancet HIV. 2020;7(5):e309–e11. Epub 2020/04/17. 32298644 10.1016/S2352-3018(20)30110-7 PMC7195084

[14] Gatechompol S, Avihingsanon A, Putcharoen O, Ruxrungtham K, Kuritzkes DR. COVID-19 and HIV infection co-pandemics and their impact: a review of the literature. AIDS Res Ther. 2021;18(1):28. Epub 2021/05/07. 33952300 10.1186/s12981-021-00335-1 PMC8097669

[15] Carpenter BS, Hanass-Hancock J, Myezwa H. Looking at antiretroviral adherence through a disability lens: a cross-sectional analysis of the intersection of disability, adherence, and health status. Disabil Rehabil. 2019:1–8. Epub 2019/01/09. 10.1080/09638288.2018.1510048 30616436

[16] Bulsara SM, Wainberg ML, Newton-John TRO. Predictors of Adult Retention in HIV Care: A Systematic Review. AIDS Behav. 2018;22(3):752–64. Epub 2016/12/19. 27990582 10.1007/s10461-016-1644-y PMC5476508

[17] Lorig KR, Sobel DS, Ritter PL, Laurent D, Hobbs M. Effect of a self-management program on patients with chronic disease. Effective clinical practice : ECP. 2001;4(6):256–62. Epub 2002/01/05. 11769298

[18] Webel AR, Lorig K, Laurent D., González V, Gifford AL, Sobel D, Living a Healthy Life with HIV; 4th Edition. Formerly Living Well with HIV & AIDS: Bull Publishing Company; 2016.

[19] Esyenbach G. Improving the quality of web surveys: the checklist for reporting results of Internet E-surveys (CHERRIES). Journal of Medicial Internet Research. 2004;6(3):e34. 10.2196/jmir.6.3.e34 PMC1550605 15471760

[20] EQUATOR Network. Enhancing the QUAlity and Transparency Of health Research. Centre for Statistics in Medicine (CSM), NDORMS, University of Oxford: UK EQUATOR Centre; 2021 [August 25, 2021]; Available from: https://www.equator-network.org/.

[21] O’Brien KK, Bayoumi AM, Solomon P, Tang A, Murzin K, Chan Carusone S, Evaluating a community-based exercise intervention with adults living with HIV: protocol for an interrupted time series study. BMJ Open. 2016;6(10):e013618. Epub 2016/11/01. 10.1136/bmjopen-2016-013618 PMC5073553 27798038

[22] Dillman DA. Mail and Internet surveys: The tailored design method−-2007 Update with new Internet, visual, and mixed-mode guide: John Wiley & Sons; 2011.

[23] Qualtrics. Qualtrics. In: Available from: http://www.qualtrics.com, editor. Provo, Utah, USA 2017.

[24] O’Brien KK, Bayoumi AM, Stratford P, Solomon P. Which dimensions of disability does the HIV Disability Questionnaire (HDQ) measure? A factor analysis. Disabil Rehabil. 2015;37(13):1193–201. 25116628 10.3109/09638288.2014.949358

[25] O’Brien KK, Solomon P, Bayoumi AM. Measuring disability experienced by adults living with HIV: assessing construct validity of the HIV Disability Questionnaire using confirmatory factor analysis. BMJ Open. 2014;4(8):e005456. Epub 2014/09/03. 10.1136/bmjopen-2014-005456 PMC4156819 25180054

[26] O’Brien KK, Bayoumi AM, King K, Alexander R, Solomon P. Community engagement in health status instrument development: experience with the HIV disability questionnaire. Progress in community health partnerships : research, education, and action. 2014;8(4):549–59. Epub 2014/01/01. 10.1353/cpr.2014.0071 25727988

[27] O’Brien KK, Bayoumi AM, Bereket T, Swinton M, Alexander R, King K, Sensibility assessment of the HIV Disability Questionnaire. Disabil Rehabil. 2013;35(7):566–77. 22816434 10.3109/09638288.2012.702848

[28] O’Brien KK, Solomon P, Bergin C, O’Dea S, Stratford P, Iku N, Reliability and validity of a new HIV-specific questionnaire with adults living with HIV in Canada and Ireland: the HIV Disability Questionnaire (HDQ). Health Qual Life Outcomes. 2015;13:124. 26263898 10.1186/s12955-015-0310-9 PMC4542093

[29] O’Brien KK, Kietrys D, Galantino ML, Parrott JS, Davis T, Tran Q, Reliability and Validity of the HIV Disability Questionnaire (HDQ) with Adults Living with HIV in the United States. Journal of the International Association of Providers of AIDS Care. 2019;18:2325958219888461. Epub 2019/11/27. 31769326 10.1177/2325958219888461 PMC6880031

[30] Brown DA, Simmons B, Boffito M, Aubry R, Nwokolo N, Harding R, Evaluation of the psychometric properties of the HIV Disability Questionnaire among adults living with HIV in the United Kingdom: A cross-sectional self-report measurement study. PloS one. 2019;14(7):e0213222. Epub 2019/07/11. 31291243 10.1371/journal.pone.0213222 PMC6619602

[31] O’Brien KK, Dagenais M, Solomon P, Worthington C, Chan Carusone S, Ibanez-Carrasco F, Use of Living Strategies among Adults Aging with HIV in Canada: Comparison by Age-Group Using Data from the HIV, Health and Rehabilitation Survey. Journal of the International Association of Providers of AIDS Care. 2018;17:2325958218774041. 29745310 10.1177/2325958218774041 PMC6748490

[32] Kroenke K, Strine TW, Spitzer RL, Williams JB, Berry JT, Mokdad AH. The PHQ-8 as a measure of current depression in the general population. J Affect Disord. 2009;114(1–3):163–73. 18752852 10.1016/j.jad.2008.06.026

[33] Do AN, Rosenberg ES, Sullivan PS, Beer L, Strine TW, Schulden JD, Excess burden of depression among HIV-infected persons receiving medical care in the united states: data from the medical monitoring project and the behavioral risk factor surveillance system. PloS one. 2014;9(3):e92842. 24663122 10.1371/journal.pone.0092842 PMC3963963

[34] Wells TS, Horton JL, LeardMann CA, Jacobson IG, Boyko EJ. A comparison of the PRIME-MD PHQ-9 and PHQ-8 in a large military prospective study, the Millennium Cohort Study. J Affect Disord. 2013;148(1):77–83. 23246365 10.1016/j.jad.2012.11.052

[35] Pearlin LI. Pearlin Mastery Scale. Journal of Health and Social Behavior. 1978;19(1):2–21. 649936

[36] R: A language and environment for statistical computing. R Foundation for Statistical Computing [database on the Internet]. GNU Operating System. 2020. Available from: https://www.R-project.org/.

[37] O’Brien KK, Hanna S, Solomon P, Worthington C, Ibanez-Carrasco F, Chan Carusone S, Characterizing the disability experience among adults living with HIV: a structural equation model using the HIV disability questionnaire (HDQ) within the HIV, health and rehabilitation survey. BMC infectious diseases. 2019;19(1):594. Epub 2019/07/10. 31286891 10.1186/s12879-019-4203-0 PMC6615082

[38] Solomon P, O’Brien K, Wilkins S, Gervais N. Aging with HIV and disability: the role of uncertainty. AIDS Care. 2014;26(2):240–5. Epub 2013/06/27. 23799874 10.1080/09540121.2013.811209

[39] Solomon P, O’Brien KK, Nixon S, Letts L, Baxter L, Gervais N. Trajectories of Episodic Disability in People Aging with HIV: A Longitudinal Qualitative Study. Journal of the International Association of Providers of AIDS Care. 2018;17:2325958218759210. 29464973 10.1177/2325958218759210 PMC6748469

[40] National Institute for Health Research (NIHR). Living with COVID19: Second Review 2021. Available from: https://evidence.nihr.ac.uk/themedreview/living-with-covid19-second-review/.

[41] Koffman J, Gross J, Etkind SN, Selman L. Uncertainty and COVID-19: how are we to respond? Journal of the Royal Society of Medicine. 2020;113(6):211–6. Epub 2020/06/11. 32521198 10.1177/0141076820930665 PMC7439590

[42] Koffman J, Gross J, Etkind SN, Selman LE. Clinical uncertainty and Covid-19: Embrace the questions and find solutions. Palliat Med. 2020;34(7):829–31. Epub 2020/06/11. 32517542 10.1177/0269216320933750

[43] Brown DA, O’Brien KK, Josh J, Nixon SA, Hanass-Hancock J, Galantino M, Six Lessons for COVID-19 Rehabilitation From HIV Rehabilitation. Phys Ther. 2020;100(11):1906–9. Epub 2020/08/02. 32737967 10.1093/ptj/pzaa142 PMC7454859

[44] Cooper TJ, Woodward BL, Alom S, Harky A. Coronavirus disease 2019 (COVID-19) outcomes in HIV/AIDS patients: a systematic review. HIV Med. 2020;21(9):567–77. Epub 2020/07/17. 32671970 10.1111/hiv.12911 PMC7405326

[45] Geretti AM, Stockdale AJ, Kelly SH, Cevik M, Collins S, Waters L, Outcomes of COVID-19 related hospitalization among people with HIV in the ISARIC WHO Clinical Characterization Protocol (UK): a prospective observational study. Clin Infect Dis. 2020. Epub 2020/10/24. 10.1093/cid/ciaa1605 PMC7665382 33095853

[46] Bhaskaran K, Rentsch CT, MacKenna B, Schultze A, Mehrkar A, Bates CJ, HIV infection and COVID-19 death: a population-based cohort analysis of UK primary care data and linked national death registrations within the OpenSAFELY platform. The lancet HIV. 2021;8(1):e24–e32. Epub 2020/12/15. 33316211 10.1016/S2352-3018(20)30305-2 PMC7773630

[47] Tesoriero JM, Swain CE, Pierce JL, Zamboni L, Wu M, Holtgrave DR, COVID-19 Outcomes Among Persons Living With or Without Diagnosed HIV Infection in New York State. JAMA network open. 2021;4(2):e2037069. Epub 2021/02/04. 33533933 10.1001/jamanetworkopen.2020.37069 PMC7859843

[48] Waterfield KC, Shah GH, Etheredge GD, Ikhile O. Consequences of COVID-19 crisis for persons with HIV: the impact of social determinants of health. BMC public health. 2021;21(1):299. Epub 2021/02/07. 33546659 10.1186/s12889-021-10296-9 PMC7863613

[49] Marziali ME, Card KG, McLinden T, Wang L, Trigg J, Hogg RS. Physical Distancing in COVID-19 May Exacerbate Experiences of Social Isolation among People Living with HIV. AIDS Behav. 2020;24(8):2250–2. Epub 2020/04/25. 32328849 10.1007/s10461-020-02872-8 PMC7178096

[50] Jones DL, Ballivian J, Rodriguez VJ, Uribe C, Cecchini D, Salazar AS, Mental Health, Coping, and Social Support Among People Living with HIV in the Americas: A Comparative Study Between Argentina and the USA During the SARS-CoV-2 Pandemic. AIDS Behav. 2021;25(8):2391–9. Epub 2021/02/26. 33630198 10.1007/s10461-021-03201-3 PMC7905200

[51] Esser S, Gelbrich G, Brockmeyer N, Goehler A, Schadendorf D, Erbel R, Prevalence of cardiovascular diseases in HIV-infected outpatients: results from a prospective, multicenter cohort study. Clinical research in cardiology : official journal of the German Cardiac Society. 2013;102(3):203–13. Epub 2012/11/03. 23117698 10.1007/s00392-012-0519-0

[52] Wang T, Fu H, Kaminga AC, Li Z, Guo G, Chen L, Prevalence of depression or depressive symptoms among people living with HIV/AIDS in China: a systematic review and meta-analysis. BMC psychiatry. 2018;18(1):160. Epub 2018/06/02. 29855289 10.1186/s12888-018-1741-8 PMC5984474

[53] Remien RH, Stirratt MJ, Nguyen N, Robbins RN, Pala AN, Mellins CA. Mental health and HIV/AIDS: the need for an integrated response. Aids. 2019;33(9):1411–20. Epub 2019/04/06. 30950883 10.1097/QAD.0000000000002227 PMC6635049

[54] O’Brien KK, Brown DA, Corbett C, Flanagan N, Solomon P, Vera JH, AIDSImpact special issue - broadening the lens: recommendations from rehabilitation in chronic disease to advance healthy ageing with HIV. AIDS Care. 2020;32(sup2):65–73. Epub 2020/03/27. 10.1080/09540121.2020.1739203 32208741

[55] Brown DA, O’Brien KK. Conceptualizing Long COVID as an Episodic Health Condition. BMJ Global Health. 2021(In Press). 10.1136/bmjgh-2021-007004 PMC8460526 34551971

[56] Power K. The COVID-19 pandemic has increased the care burden of women and families. Sustainability: Science, Practice, and Policy. 2020;16(1):67–73.

[57] Wenham C, Smith J, Morgan R, Gender, Group C-W. COVID-19: the gendered impacts of the outbreak. Lancet. 2020;395(10227):846–8. Epub 2020/03/11. 32151325 10.1016/S0140-6736(20)30526-2 PMC7124625

[58] Torjesen I. Covid-19: Middle aged women face greater risk of debilitating long term symptoms. Bmj. 2021;372:n829. Epub 2021/03/27. 33766927 10.1136/bmj.n829

