# Supplementary material for: Disability and Self-care Living Strategies Among Adults Living With HIV During the COVID-19 Pandemic
Source: Res Sq. 2021 Sep 14:rs.3.rs-868864. Preprint. [Version 1] doi: 10.21203/rs.3.rs-868864/v1 (PMC8452102; doi:10.21203/rs.3.rs-868864/v1)
Supplement: Supplement 2 [file 5d5165e920460e7bc4f01bed.pdf]

Additional File 1 - PHQ8 (Mental Health) and Mastery Scores – Pre and During the COVID-19 Pandemic

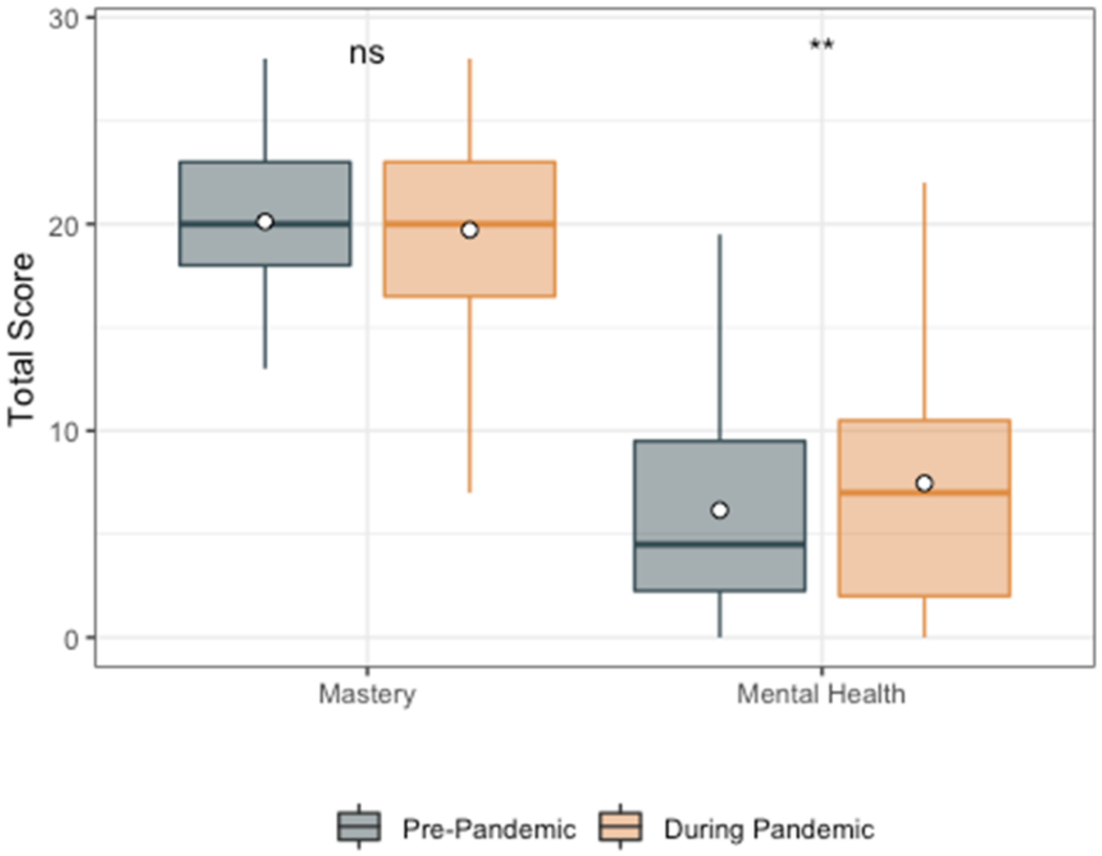

Figure Legend: Significance of paired t-tests is shown as follows: *** p<0.001, ** 0.001 < p <= 0.01, * 0.01< p <= 0.05 and ns indicates p>0.05. n=51 participants. Boxplots indicate median line and inter-quartile range, whiskers indicate 1.5 x IQR. Mean is shown as a circle.

PHQ8 and Mastery Scores: Pre versus During COVID-19 Pandemic (n=51 participants)

Questionnaire	Spearman Correlation coefficients	Pre-Pandemic Mean (sd) Scores	During Pandemic Mean (sd) Scores	Mean (sd) Change in Scores	Paired T-test	P value
PHQ8 Score	0.6	6.2 (4.8)	8.0 (6.4)	1.9 (5.0)	-2.69	0.010**
Mastery Score	0.6	20.1 (3.5)	19.3 (4.5)	-0.8 (3.9)	-1.56	0.126

LEGEND: Patient Health Questionnaire (PHQ8) (score range: 0-24) of which scores of ≥5, ≥10, and ≥20 indicate mild, moderate, and severe depression, respectively; Mastery Scale score range from 7 to 28; higher scores indicate greater mastery; degrees of freedom: 50; *** indicates p<0.001, ** indicates 0.001 < p <= 0.01, * indicates 0.01< p <= 0.05.
