# Supplementary material for: Disability and Self-care Living Strategies Among Adults Living With HIV During the COVID-19 Pandemic
Source: Res Sq. 2021 Sep 14:rs.3.rs-868864. Preprint. [Version 1] doi: 10.21203/rs.3.rs-868864/v1 (PMC8452102; doi:10.21203/rs.3.rs-868864/v1)
Supplement: Supplement 3 [file 17d00bbbd60656dcea726b45.pdf]

Additional File 2 - Participant Change in PHQ8 Classification Category of Depression Pre and During the Pandemic (n=51 participants)

Pre-Pandemic	During Pandemic				
	None	Mild	Moderate	Severe	Total
None	15	9	2	0	26
Mild	2	5	4	1	12
Moderate	1	3	5	4	13
Severe	0	0	0	0	0
Total	18	17	11	5	51

LEGEND: PHQ8 Questionnaire (score range: 0-24) of which scores of ≥5, ≥10, and ≥20 indicate mild, moderate, and severe depression, respectively.
Of the 51 participants, 25 (49%) remained stable (orange highlight), 20 (39%) reported increased depression (blue highlight) and 6 (12%) reported less depression (green).
