# Supplementary material for: Disability and Self-care Living Strategies Among Adults Living With HIV During the COVID-19 Pandemic
Source: Res Sq. 2021 Sep 14:rs.3.rs-868864. Preprint. [Version 1] doi: 10.21203/rs.3.rs-868864/v1 (PMC8452102; doi:10.21203/rs.3.rs-868864/v1)
Supplement: Supplement 4 [file ca2a969879b26f9a2840f76a.pdf]

Additional File 4 – Associations between Disability Severity and Self-Care Living Strategies Use during the Pandemic (n=63 participants)

**Living Strategy**	**Living Strategy Component of Episodic Disability Framework**	**Mental-Emotional** Spearman Correlation Coefficient (95% CI)	**Uncertainty** Spearman Correlation Coefficient (95% CI)	**Day to Day** Spearman Correlation Coefficient (95% CI)	**Social** Spearman Correlation Coefficient (95% CI)	**Cognitive** Spearman Correlation Coefficient (95% CI)
I focus on things such as work, friends and activities.	Maintaining Sense of Control (maintain focus / establishing purpose)	-0.47 (-0.65, -0.24)	--	--	--	--
I maintain a good balance of activity in my life.	Maintaining Sense of Control (maintain focus / establishing purpose)	-0.51 (-0.68, -0.29)	--	--	--	--
I try to stick to daily structure or routine	Maintaining Sense of Control (maintain life balance)	-0.46 (-0.64, -0.23)	--	--	--	--
I consider myself healthy.	Attitudes and Beliefs (positive outlook)	-0.60 (-0.75, -0.39)	-0.54 (-0.70, -0.32)	-0.57 (-0.73, -0.36)	-0.50 (-0.68, -0.28)	--
I accept and value who I am – the good and the bad.	Attitudes and Beliefs (positive outlook)	-0.47 (-0.65, -0.24)	-0.50 (-0.68, -0.28)	--	-0.52 (-0.69, -0.29)	--
I have a positive outlook on life and use hope and optimism.	Attitudes and Beliefs (positive outlook)	-0.54 (-0.71, -0.33)	--	-0.46 (-0.65, -0.23)	-0.47 (-0.65, -0.24)	--
I choose to believe I can survive and overcome any challenges.	Attitudes and Beliefs (positive outlook)	-0.52 (-0.69, -0.30)	--	-0.53 (-0.70, -0.31)	-0.47 (-0.65, -0.24)	--
I feel hopeless.*	Attitudes and Beliefs (positive outlook)	0.62 (0.42, 0.76)	--	--	0.46 (-0.23, 0.65)	0.48 (0.25, 0.66)

*negative strategy; correlation coefficients all significant p<0.0001.

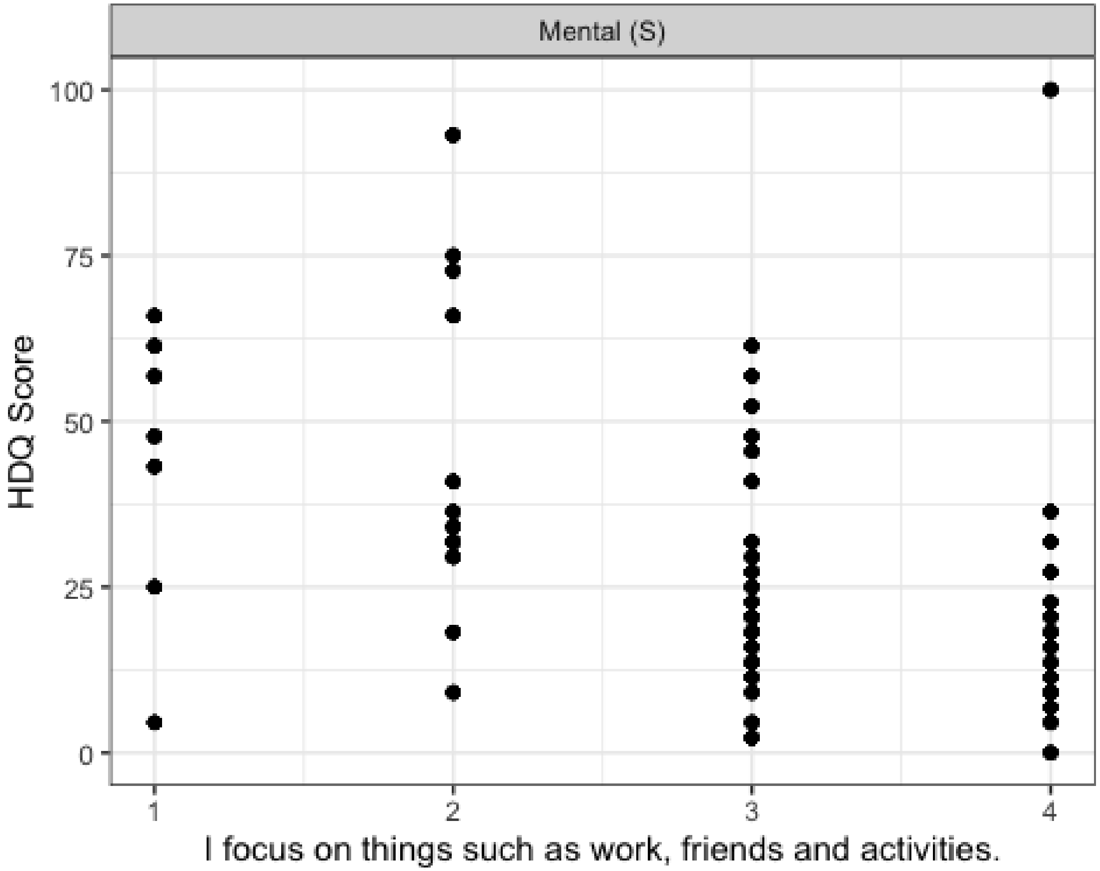

HDQ Variable	spearman	95% CI
Mental (S)	-0.47	-0.65, -0.24

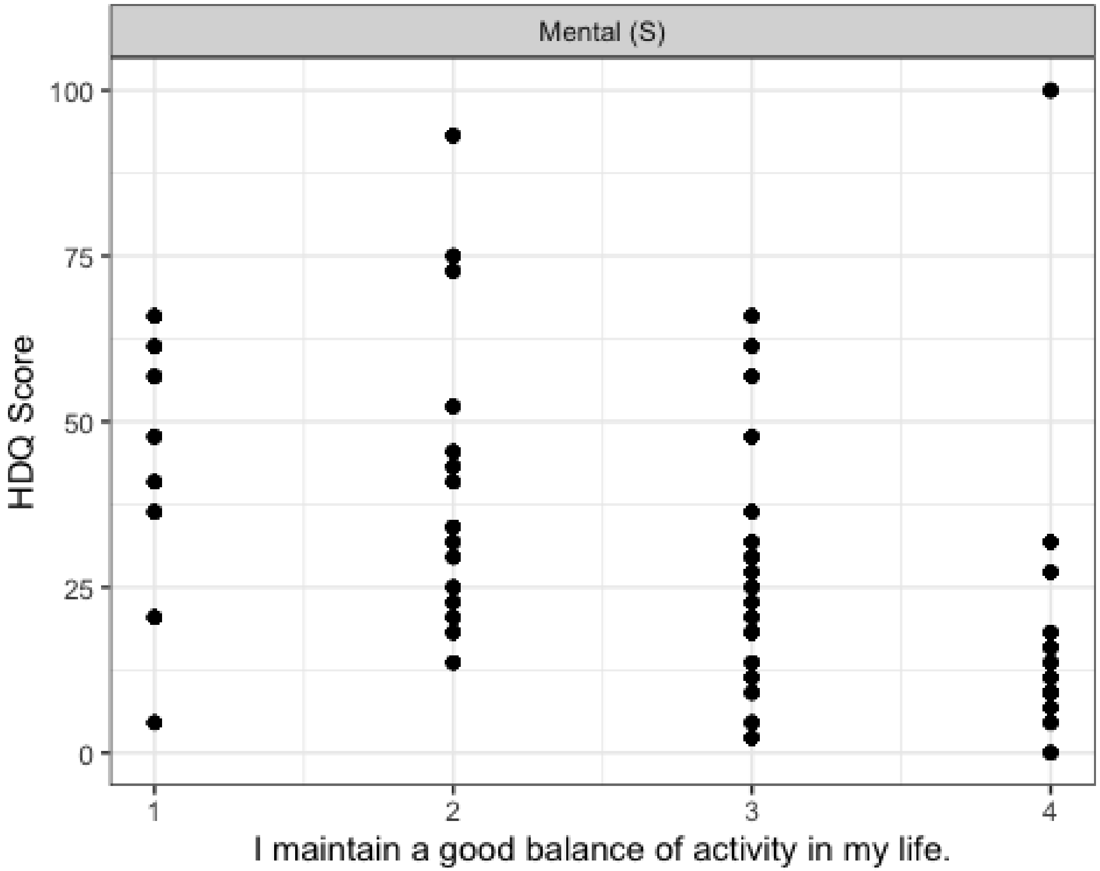

HDQ Variable	spearman	95% CI
Mental (S)	-0.51	-0.68, -0.29

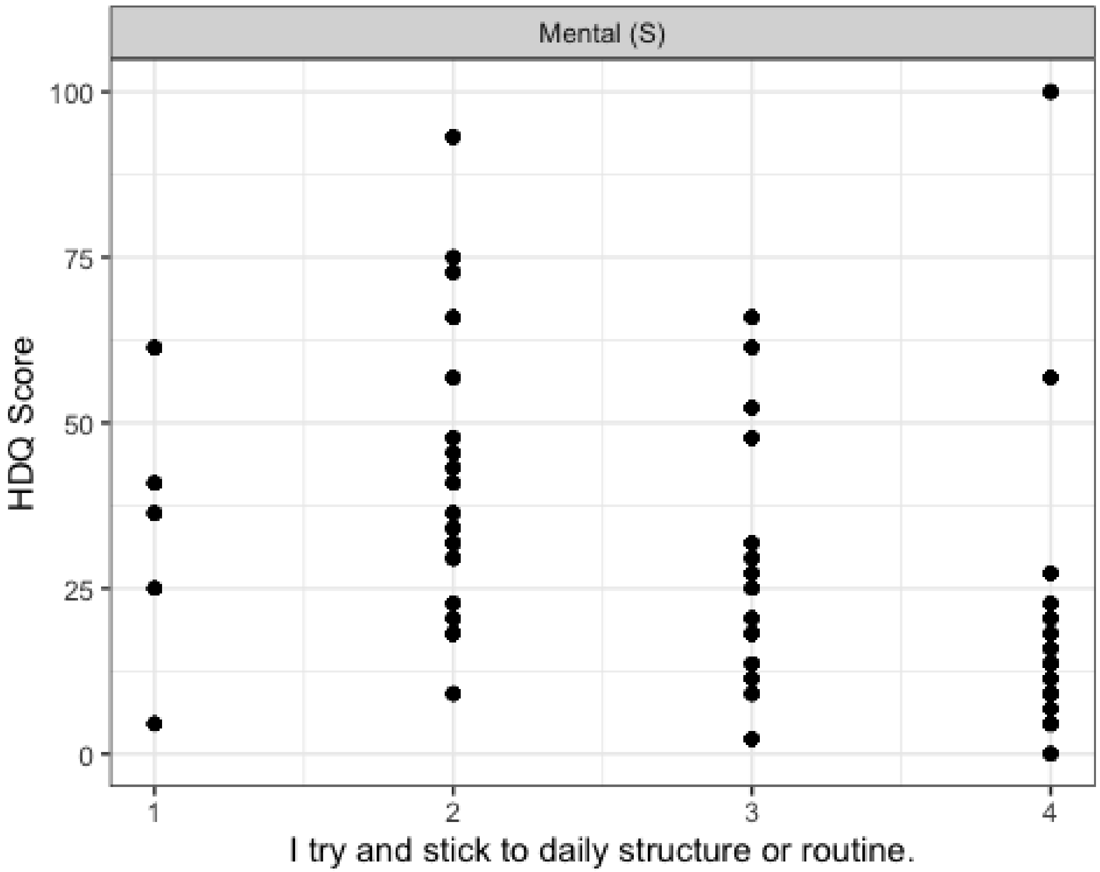

HDQ Variable	spearman	95% CI
Mental (S)	-0.46	-0.64, -0.23

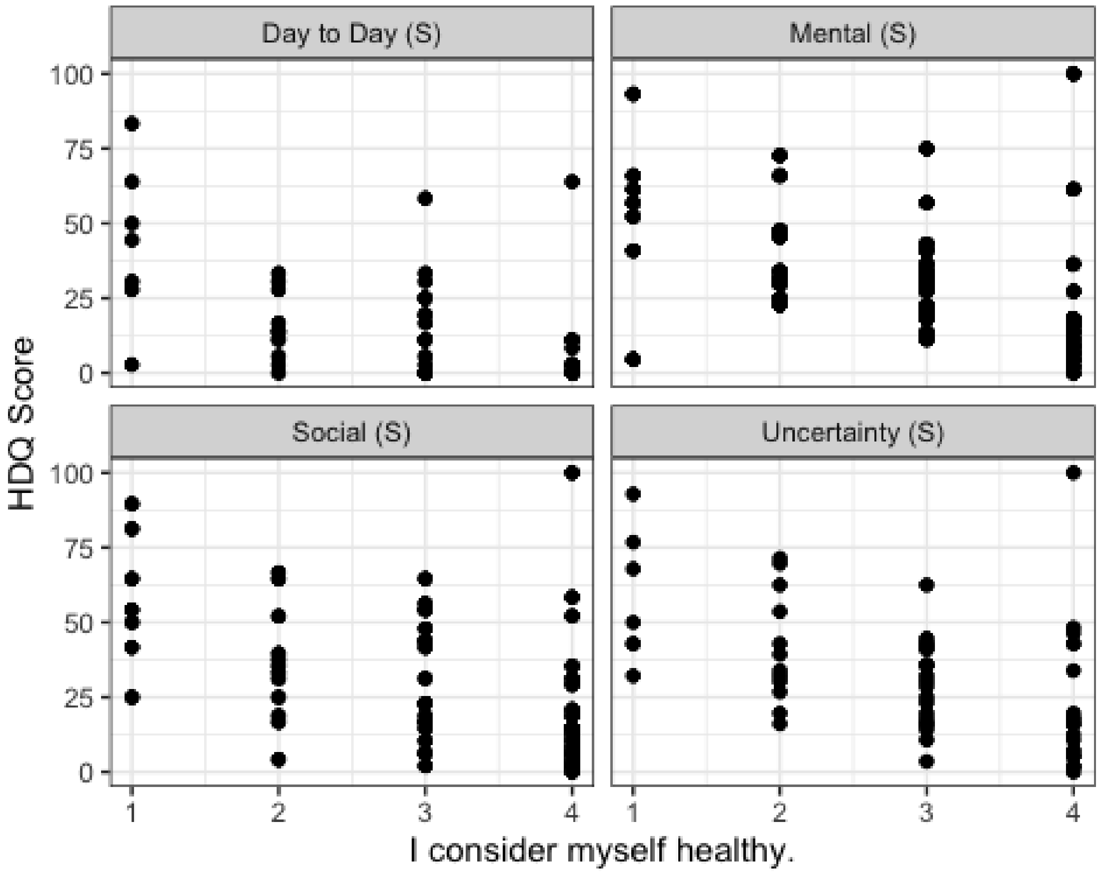

HDQ Variable	spearman	95% CI
Mental (S)	-0.60	-0.75, -0.39
Uncertainty (S)	-0.54	-0.7, -0.32
Day to Day (S)	-0.57	-0.73, -0.36
Social (S)	-0.50	-0.68, -0.28

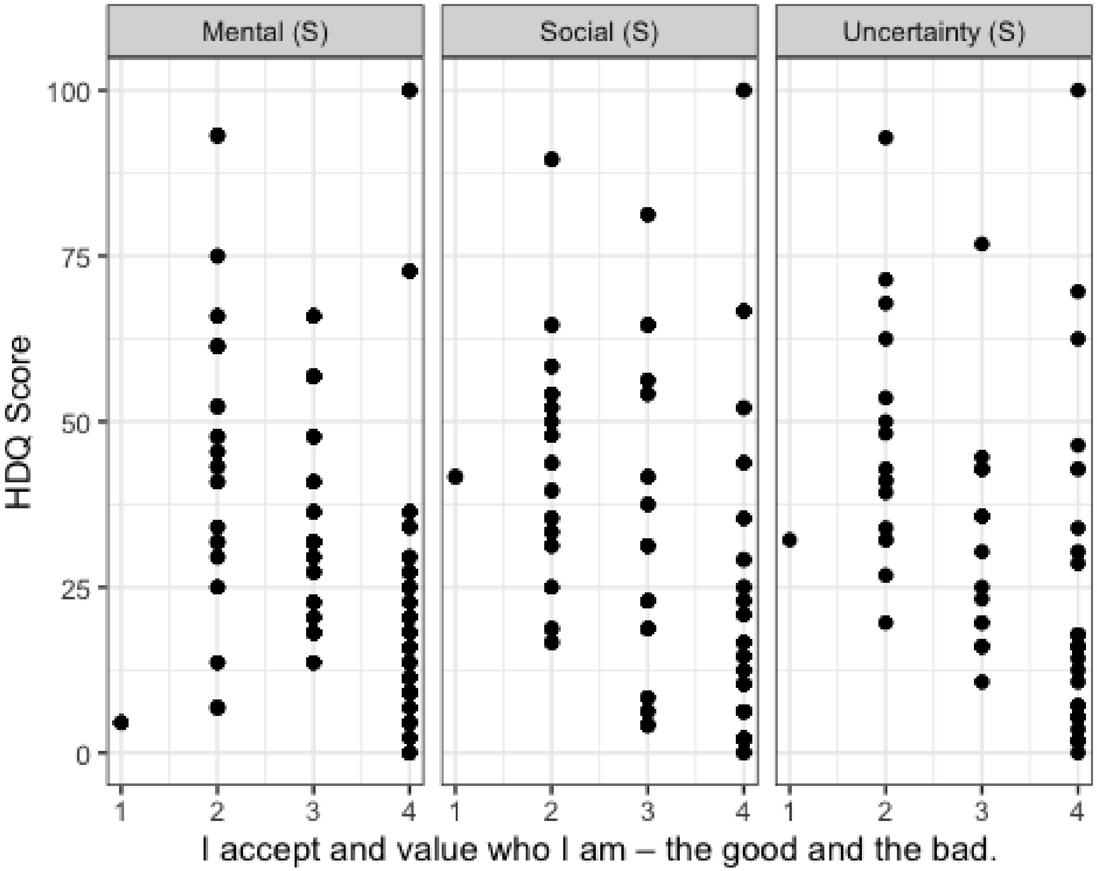

HDQ Variable	spearman	95% CI
Mental (S)	-0.47	-0.65, -0.24
Uncertainty (S)	-0.50	-0.68, -0.28
Social (S)	-0.52	-0.69, -0.29

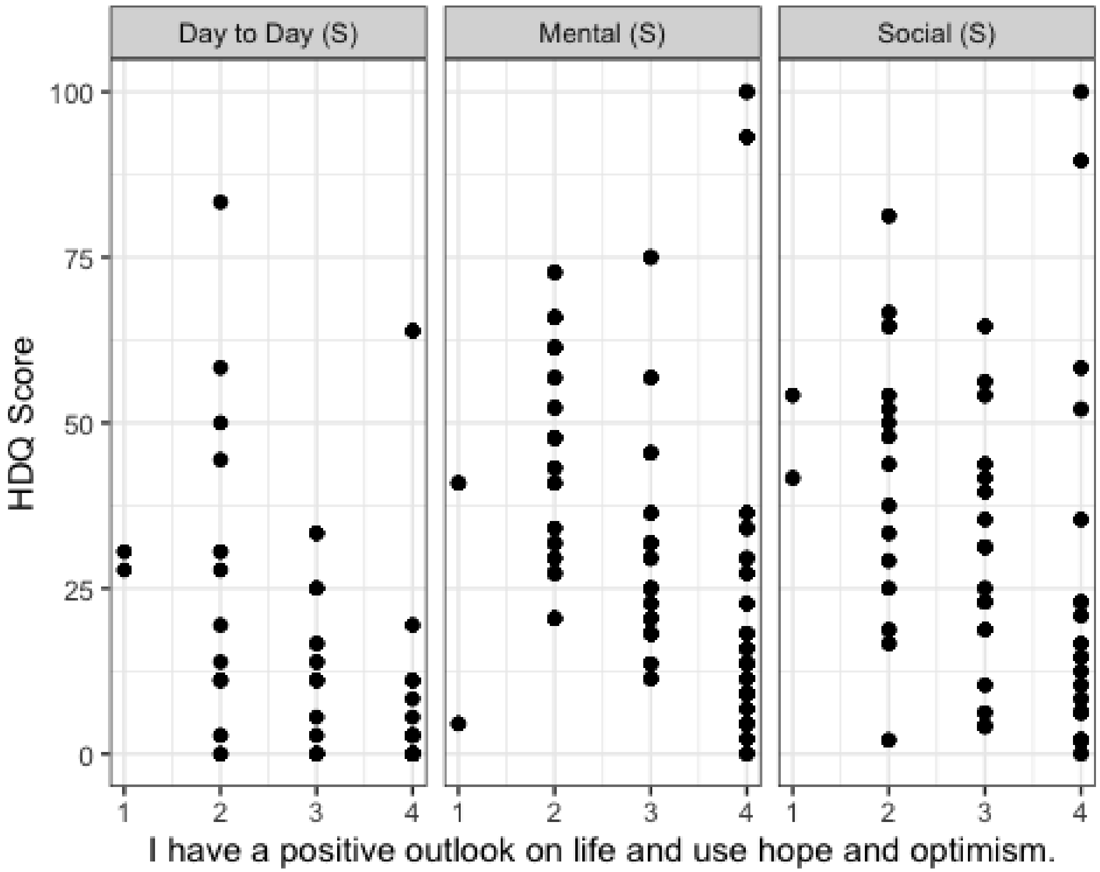

HDQ Variable	spearman	95% CI
Mental (S)	-0.54	-0.71, -0.33
Day to Day (S)	-0.46	-0.65, -0.23
Social (S)	-0.47	-0.65, -0.24

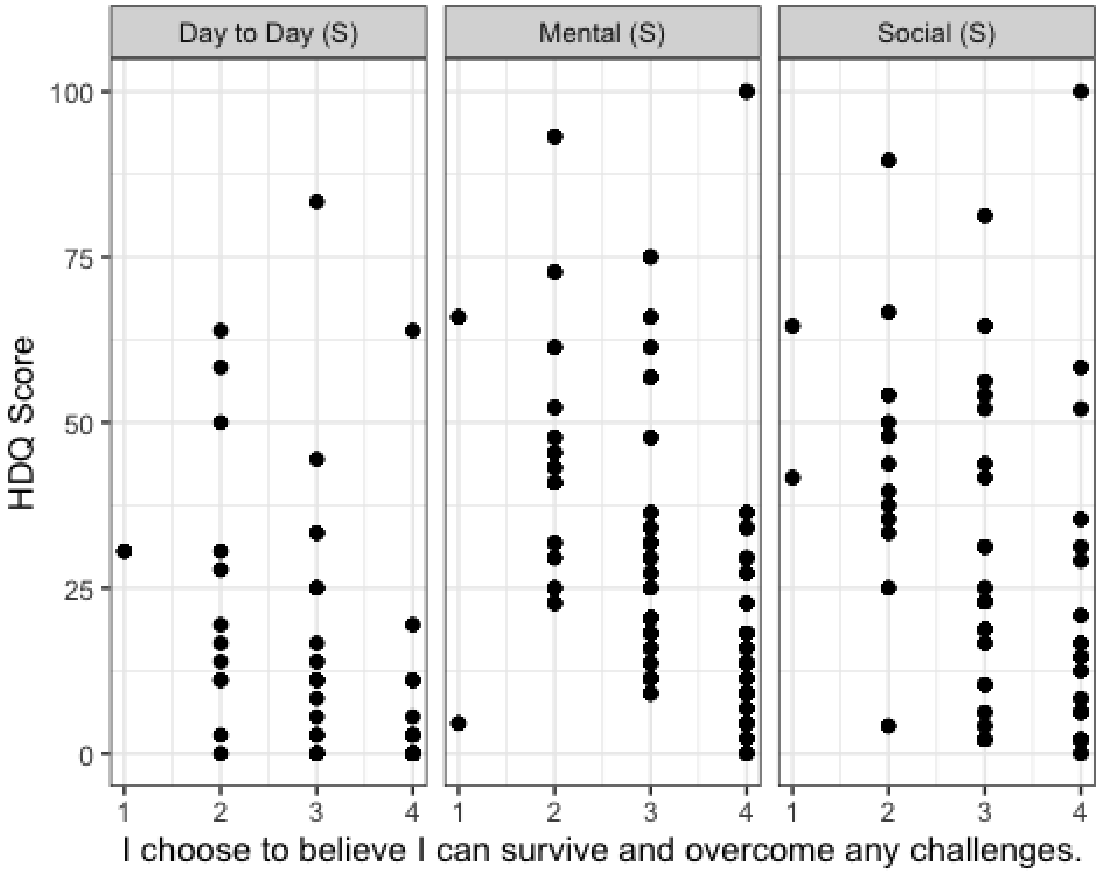

HDQ Variable	spearman	95% CI
Mental (S)	-0.52	-0.69, -0.3
Day to Day (S)	-0.53	-0.7, -0.31
Social (S)	-0.47	-0.65, -0.24

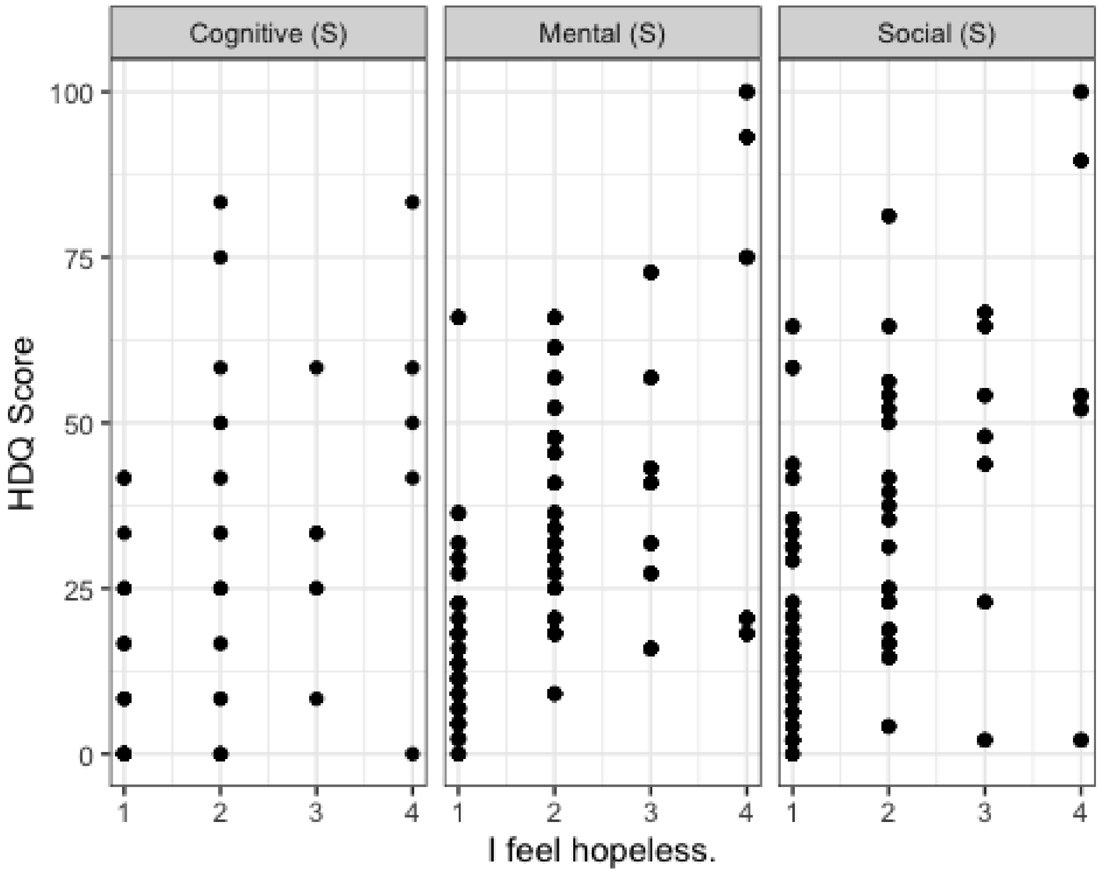

HDQ Variable	spearman	95% CI
Cognitive (S)	0.48	0.25, 0.66
Mental (S)	0.62	0.42, 0.76
Social (S)	0.46	0.23, 0.65
